# Self-Decoration of PtNi Alloy Nanoparticles on Multiwalled Carbon Nanotubes for Highly Efficient Methanol Electro-Oxidation

**DOI:** 10.1007/s40820-016-0096-2

**Published:** 2016-07-01

**Authors:** Yu-Yan Zhou, Chang-Hai Liu, Jie Liu, Xin-Lei Cai, Ying Lu, Hui Zhang, Xu-Hui Sun, Sui-Dong Wang

**Affiliations:** 1 Institute of Functional Nano & Soft Materials (FUNSOM), Jiangsu Key Laboratory for Carbon-Based Functional Materials & Devices, Soochow University, Suzhou, 215123 Jiangsu People’s Republic of China; 2 School of Materials Science & Engineering, Jiangsu Collaborative Innovation Center of Photovoltaic Science and Engineering, Changzhou University, Changzhou, 213164 Jiangsu People’s Republic of China

**Keywords:** PtNi nanoparticles, Multiwalled carbon nanotubes, Methanol electro-oxidation

## Abstract

**Abstract:**

A simple one-pot method was developed to prepare PtNi alloy nanoparticles, which can be self-decorated on multiwalled carbon nanotubes in [BMIm][BF_4_] ionic liquid. The nanohybrids are targeting stable nanocatalysts for fuel cell applications. The sizes of the supported PtNi nanoparticles are uniform and as small as 1–2 nm. Pt-to-Ni ratio was controllable by simply selecting a PtNi alloy target. The alloy nanoparticles with Pt-to-Ni ratio of 1:1 show high catalytic activity and stability for methanol electro-oxidation. The performance is much higher compared with those of both Pt-only nanoparticles and commercial Pt/C catalyst. The electronic structure characterization on the PtNi nanoparticles demonstrates that the electrons are transferred from Ni to Pt, which can suppress the CO poisoning effect.

**Graphical Abstract:**

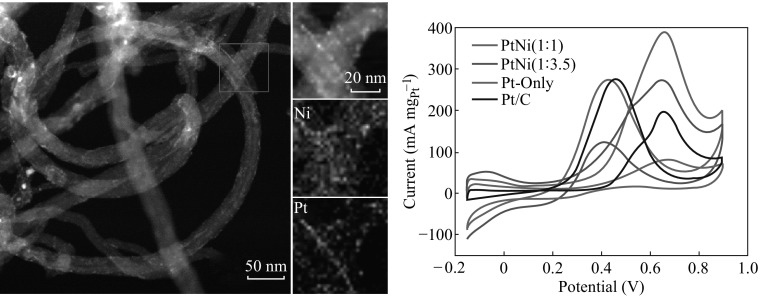

**Electronic supplementary material:**

The online version of this article (doi:10.1007/s40820-016-0096-2) contains supplementary material, which is available to authorized users.

## Introduction

To satisfy increasing demands in energy and overcome issues of environment pollution, researches on sustainable and renewable energy which may replace traditional fossil fuels have become hot topics. Direct methanol fuel cells (DMFCs), a green power sources in vehicles and portable devices, have gained wide attentions [1–3]. Methanol as fuel has numerous advantages: simplicity in handling, storage, and transport; low cost and renewability because it can be easily obtained from fermentation of agricultural products [4–6]. The efficiency of methanol electro-oxidation is limited by poor kinetics and methanol crossover [6, 7], and thus the development of anode catalysts with high catalytic performance is in an urgent need to achieve efficient DMFCs. Pt is the most widely used catalyst for electrochemical oxidation of methanol. However, during the electro-oxidation process, CO molecules are produced as the intermediate, which could be adsorbed on the Pt surface and hardly be removed. As a consequence, the surface poisoning of Pt by CO will suppress the catalytic activity, and this issue must be addressed for the realization of efficient and stable Pt catalysts [7, 8].

The methanol electro-oxidation on Pt is considered to follow a three-step process. The first is the adsorption of methanol. Subsequently, Pt breaks the C–H bonds of methanol and then CO molecules will adsorb on the Pt surface, which is regarded as the dehydrogenation step. Eventually, CO is oxidized with the assistance of oxygen-containing species (e.g., –OH) formed on Pt [9, 10]. However, the oxygen-containing species are often formed on the Pt surface at high potentials [>0.7 V vs reversible hydrogen electrode (RHE)] [7, 10, 11]. Therefore, at the state of low potentials, the adsorbed CO molecules are very difficult to be removed, which results in poor activity of Pt catalysts.

Introducing a second or third metal, such as Ni, Au, Ru, Sn, Co, or Cu [12–19], is an effective way to liberate the Pt surface from the CO adsorption. In this type of bimetallic systems, Pt plays a key role for the adsorption of methanol and dehydrogenation. On the other hand, the second metal could supply oxygen-containing species as the promoter for the CO oxidation at low potentials [20]. The second metal could also modify the electronic structure of Pt by transferring electrons from the second metal to Pt and thus weakening the Pt–CO bonding energy [21, 22]. The binary and ternary Pd-based catalysts were also along the same path [23, 24]. Furthermore, the addition of a second metal will reduce the consumption of Pt, which is in favor of minimizing the catalyst cost.

Among bimetallic catalysts, PtNi nanoparticles (NPs) in particular are promising materials for methanol electro-oxidation since Ni is more economic than other metals [14, 22, 25]. PtNi catalysts with enhanced activity have been prepared by the microwave-assisted polyol reduction, and the enhancement in catalytic activity is attributed to their electronic structure modification [7]. The X-ray photoelectron spectroscopy (XPS) results show that the Pt 4f peak for PtNi is shifted to the lower binding energy, demonstrating the electron transfer from Ni to Pt [7]. The density functional theory (DFT) studies further indicate that incorporating Ni will induce an upshift in the *d*-band center of Pt, leading to a weakening of the interaction between Pt and CO [14, 26].

The reduction of metal ions in solution is a typical approach to synthesize metal and alloy NPs [25, 27], which often involves chemical additives and/or byproducts, such as surfactants or polymers used to stabilize metal NPs. However, the introduction of the organic materials would block the active surface of metal and alloy NPs to some extent. To realize an additive/byproduct free approach, we develop a room-temperature ionic liquid (RTIL)-assisted sputtering method to prepare supported metal and alloy NPs. RTILs herein play an important role for uniform dispersion of nanosupports and stabilization of small-sized metal NPs. In this method, metal is directly sputtered onto a suspension mixing RTILs and nanosupports [28–31], where metal NPs may nucleate on the RTIL surface, and then diffuse into the suspension and finally self-decorate on the nanosupports [24, 32–34]. This method has been extended to prepare supported bimetallic NPs, such as AgPd and AgAu NPs decorated on graphene, and PdAu NPs decorated on carbon or TiO_2_ nanosupports [31, 35–37].

The RTIL-assisted sputtering is utilized to synthesize PtNi alloy NPs decorated on multiwalled carbon nanotubes (MWNTs), which possess large surface area, high conductivity, and good chemical stability. In particular, the PtNi NPs were prepared straightforwardly by sputtering a PtNi alloy target, and thus the atomic ratio of Pt to Ni can be easily controlled by changing the alloy target. The self-decoration on MWNTs is demonstrated to be an effective way to stabilize the PtNi alloy NPs, which have small sizes of only a few nanometers and uniform distribution on the nanosupport. Upon the employment of an appropriate Pt-to-Ni ratio, the supported PtNi NPs showed high catalytic activity and high stability for methanol electro-oxidation. The catalytic performance of the alloy NPs is superior to those of the Pt-only NPs and the commercial Pt/C catalyst.

## Experimental

### Preparation of Pt/PtNi-NP-MWNT Hybrids

RTIL, 1-butyl-3-methylimidazolium tetrafluoroborate ([BMIm][BF_4_]), purity >99 %), was purchased from Shanghai Cheng-Jie Chemical and dried in vacuum for 24 h before using. MWNTs with diameters ranging from 30 to 50 nm were purchased from Nanjing XFNANO Materials Tech. The commercial Pt/C catalyst was purchased from Alfa Aesar.

Firstly, 10 mg MWNTs was fully dispersed into 1.5 mL [BMIm][BF_4_], with ultrasonication for 30 min to produce the [BMIm][BF_4_]-MWNT suspension. A stainless steel pot containing the suspension was placed into the sputtering chamber. A Pt or PtNi alloy target was used to sputter Pt or PtNi, respectively, onto the suspension for 15 min in a desktop direct-current sputtering system (Quorum Technologies). Two PtNi alloy targets were selected: one is Pt rich and the other is relatively Ni rich. The actual Pt-to-Ni ratio of the PtNi NPs was estimated by inductively coupled plasma atomic emission spectroscopy (ICP-AES, Vista-MPX), and two Pt-to-Ni atomic ratios of 1:1 and 1:3.5 were obtained. During the sputtering, the Ar working pressure and deposition rate were kept at ~0.01 mbar and 0.2 Å s^−1^, respectively. Eventually, the Pt/PtNi-NP-MWNT hybrids were separated from [BMIm][BF_4_] by centrifugation, and the supernatant liquid was decanted after the centrifugation. The hybrids were then washed by acetone several times to completely remove residual [BMIm][BF_4_]. To liberate the catalysts from possible surface contamination, the hybrids were annealed at ~300 °C for 1 h in H_2_-Ar environment (~2.5 mbar, H_2_:Ar = 1:9).

### Characterization of Pt/PtNi-NP-MWNT Hybrids

The metal loading and composition of the hybrids were analyzed by ICP-AES, and the measured Pt loading is shown in Table 1. The Pt-based catalysts were sampled by treating with aqua regia. MWNTs cannot be dissolved, and then were discarded by centrifugation. Pt and Ni were dissolved and left in solution for the ICP-AES measurements. To confirm the validity of the ICP-AES results, a commercial Pt/C catalyst with known metal loading of 20 wt% was also measured as reference. The microscopic structures of the hybrids were characterized with high-resolution transmission electron microscopy (HRTEM, FEI Quanta FRG 200F) and high-angle annular dark-field scanning TEM (HAADF-STEM). The electronic structures of the hybrids were characterized with XPS (Kratos Axis Ultra DLD, monochromatic Al *Kα* source). The X-ray absorption near-edge structure (XANES) measurements of the hybrids were performed at the Taiwan light source (TLS).

**Table 1:** Metal composition of different catalysts measured by ICP-AES

Catalyst	Metal loading [wt%]		Atomic ratio of Pt:Ni
	Pt	Ni	
Pt/C	19.22	–	–
Pt-only	7.74	–	–
PtNi (1:1)	6.76	1.91	1:1
PtNi (1:3.5)	3.35	3.48	1:3.5

### Electrochemical Measurements

The electrochemical measurements were carried out using CHI660E with three-electrode configuration. A carbon electrode was used as the counter electrode, and an Ag/AgCl electrode was used as the reference electrode with respect to which potentials were measured. On the other hand, a catalyst-coated glassy carbon (GC) electrode (3 mm in diameter) was employed as the working electrode. Before the preparation of the working electrode, the GC electrode was polished using an alumina slurry of 0.05 μm. Subsequently, the GC electrode was ultrasonically washed in a mixed solution of ethanol and deionized water of 1:1 in volume, and then dried at room temperature.

The catalyst suspensions were obtained by fully dispersing 3 mg catalyst in a mixed solution of 1 mL ethanol and 7 μL Nafion solution (5 wt% purchased from Aldrich) with ultrasonication for 1 h. The working electrode was prepared by dropping 10 μL catalyst ink onto the GC electrode and then drying it in air, yielding a working electrode with a catalyst loading of 0.42 mg cm^−2^. The actual mass of Pt in the catalyst ink were 5.96, 1.75, 1.39, and 0.85 μg for Pt/C, Pt-only, PtNi (1:1) and PtNi (1:3.5), respectively. Cyclic voltammogram (CV) was carried out with typical parameters, at a scan rate of 50 mV s^−1^ in a solution of 1 M CH_3_OH + 0.5 M H_2_SO_4_, to evaluate the catalytic activity for methanol electro-oxidation.

## Results and Discussion

### Microscopic Structures

The metal composition and loading of the as-prepared nanocatalysts and commercial Pt/C catalyst were measured by ICP-AES, and the results are summarized in Table 1. The sputtering of a Pt-rich alloy target produces the PtNi NPs with measured Pt-to-Ni ratio of about 1:1. Ni appears to be easier to decorate onto MWNTs in [BMIm][BF_4_] compared with Pt [38, 39], presumably due to the fact that Ni is more active than Pt. On the other hand, employing a relatively Ni-rich alloy target leads to the PtNi NPs with measured Pt-to-Ni ratio of about 1:3.5. The two groups of PtNi samples with different Pt-to-Ni ratios and the pure Pt hybrid as the control sample were denoted as the PtNi (1:1), PtNi (1:3.5), and Pt-only NPs, respectively.

**Fig. 1:**
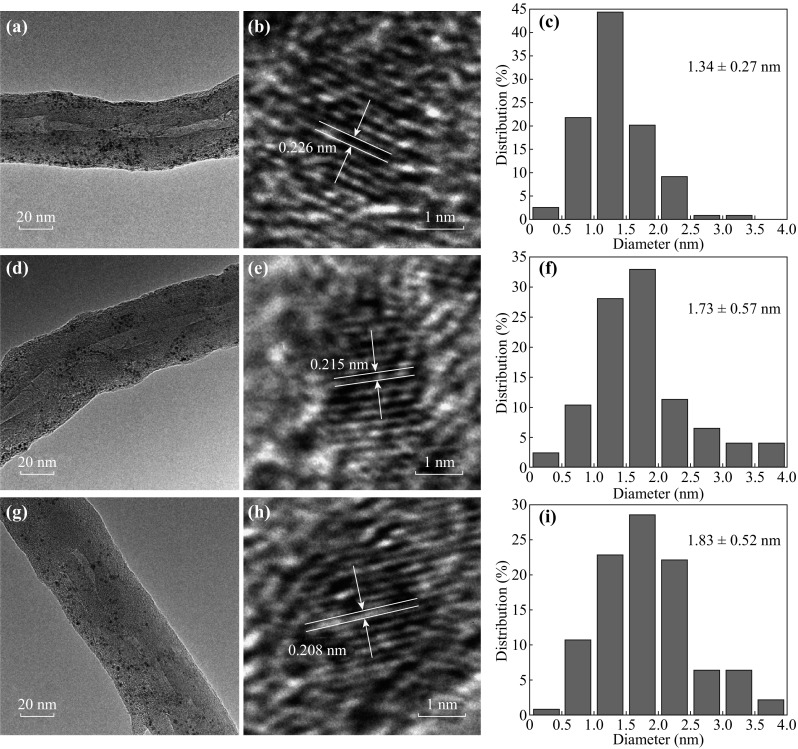
TEM images, HRTEM images, and NP size distributions in Pt/PtNi-NP-MWNT hybrids: **a**–**c** Pt-only NPs; **d**–**f** PtNi (1:1) NPs; and **g**–**i** PtNi (1:3.5) NPs

Figure 1a, d, g shows the TEM images of the Pt/PtNi-NP-MWNT hybrids, which exhibit uniform size distribution and good dispersion of NPs on MWNTs. The average NP diameters are 1.34, 1.73, and 1.83 nm for the Pt-only, PtNi (1:1), and PtNi (1:3.5) NPs, respectively. Upon addition of Ni, the NP size is only slightly increased, and it is much smaller than that in the commercial Pt/C catalyst (average Pt NP diameter of 4.04 nm, see Fig. S1 in Supporting Information). Figure 1b, e, h shows the typical HRTEM images of the Pt-only, PtNi (1:1), and PtNi (1:3.5) NPs, respectively. The interplanar spacing of 0.226 nm for Pt-only matches well with the (111) plane of face-centered-cubic (fcc) Pt [40]. Upon introduction of Ni, the spacing value is gradually decreased, e.g., the PtNi (1:1) NPs exhibit the interplanar spacing of 0.217 nm and the PtNi (1:3.5) NPs show the one of 0.208 nm. The lattice contraction suggests that the PtNi NPs are in an alloy form [41].

**Fig. 2:**
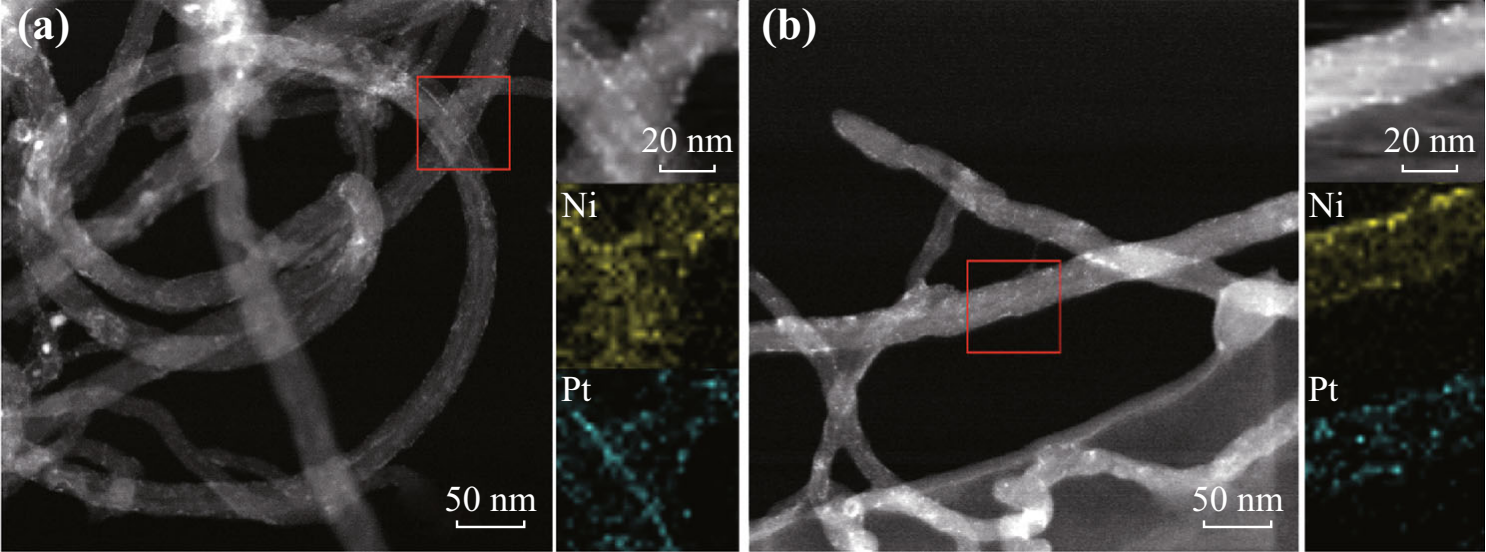
HAADF-STEM images of **a** PtNi (1:1) NPs and **b** PtNi (1:3.5) NPs on MWNTs, where elemental mapping of Pt and Ni is shown

The HAADF-STEM technique was utilized to verify the spatial distribution of Pt and Ni in the PtNi-NP-MWNT hybrids, and the results are shown in Fig. 2. The dark-field images show that the PtNi NPs are decorated on the entire surface of MWNTs. The STEM mapping demonstrates the uniformity and overlapping in spatial distribution for both Pt and Ni, supporting the alloying of the PtNi NPs regardless of Pt-to-Ni ratio. The microscopic structure results indicate that the PtNi NPs can self-decorate onto MWNTs in [BMIm][BF_4_] and maintain the small NP size and uniform NP distribution.

### Electronic Structures

**Fig. 3:**
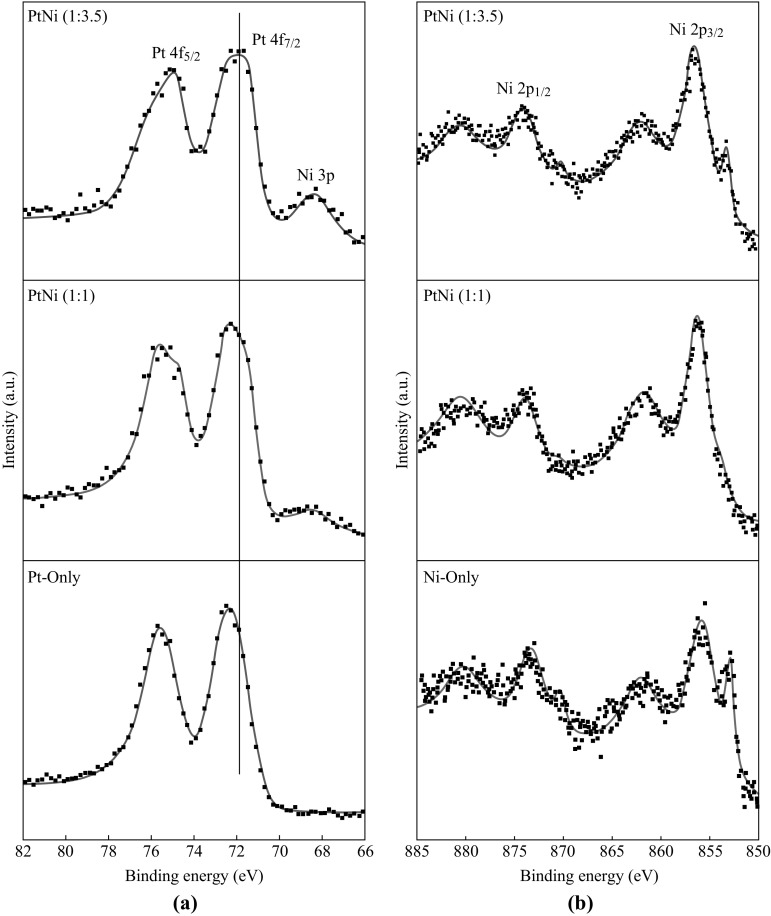
XPS spectra of Pt/Ni/PtNi-NP-MWNT hybrids: **a** Pt 4*f* region and **b** Ni 2*p* region

XPS was used to analyze the chemical states of the Pt/PtNi-NP-MWNT hybrids, and the XPS Pt 4*f* spectra are shown in Fig. 3a, where a vertical reference line is present. Compared with the Pt 4*f*_7/2_ peak of bulk Pt at binding energy of about 71 eV, there is a significant shift of the Pt 4*f*_7/2_ peak to higher binding energy for the hybrids. The increase in binding energy may be due to the small NP size and the interaction of Pt with the support [42, 43]. Upon the addition of Ni, the Pt 4*f*_7/2_ peak of the PtNi (1:1) NPs is negatively shifted compared to that of the Pt-only NPs. With increasing the proportion of Ni, the Pt 4*f*_7/2_ peak of the PtNi (1:3.5) is further shifted to lower binding energy. Such shifts in binding energy suggest the electron transfer from Ni to Pt arising from the PtNi alloy formation [40].

On the other hand, the XPS Ni 2*p* spectra of the hybrids are shown in Fig. 3b for comparison. In all the spectra, the Ni oxidized state (e.g., at binding energy of about 856 eV) together with a broad satellite peak at higher binding energy account for the vast majority. The Ni metallic state at binding energy of about 853 eV is present as well, whereas it is gradually disappeared with increasing the Pt proportion. The XPS Ni features are consistent with the Pt ones, indicating an electron loss of Ni and an electron gaining of Pt in the PtNi NPs.

**Fig. 4:**
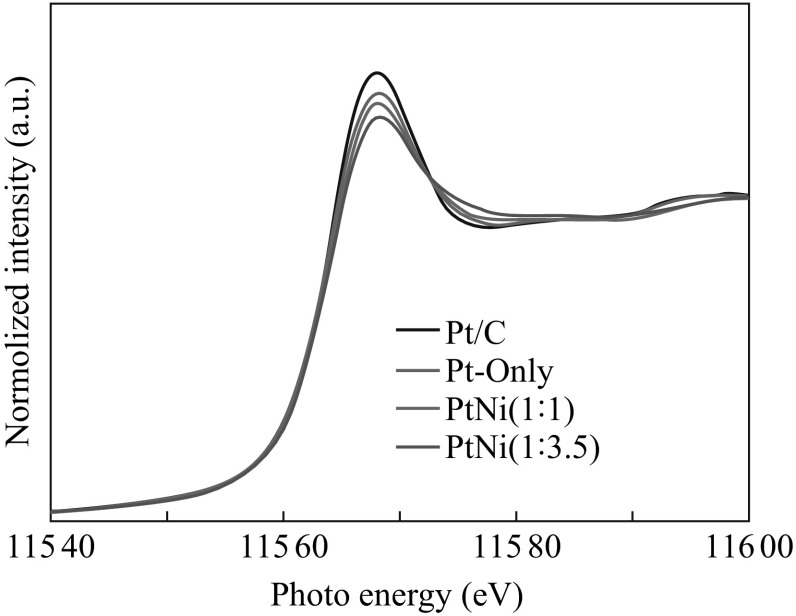
Pt *L*_3_-edge XANES spectra of Pt/PtNi-NP-MWNT hybrids and commercial Pt/C catalyst

To make a double check on the electronic structures of the PtNi NPs, the Pt *L*_3_-edge XANES spectra were measured at the TLS and the results are shown in Fig. 4. The absorption peak at about 11.57 keV corresponds to the electronic transition from Pt 2*p* to 5*d*, and the absorption edge called as the whiteline can reflect the density of unoccupied states in the *d*-band of Pt. The whiteline intensity decreases with decrease of unoccupied states in 5*d* band [7]. As demonstrated in Fig. 4, the whiteline intensity for the Pt/PtNi-NP-MWNT hybrids is lower than that for Pt/C. Furthermore, the whiteline intensity for the PtNi NPs is lower compared with the Pt-only NPs, in particular upon increasing the proportion of Ni. The XANES results imply that the PtNi NPs possess less unoccupied states in the *d*-band of Pt due to the electron transfer from Ni to Pt, in good agreement with our argument of the PtNi alloy formation.

### Electrochemical Performance

**Fig. 5:**
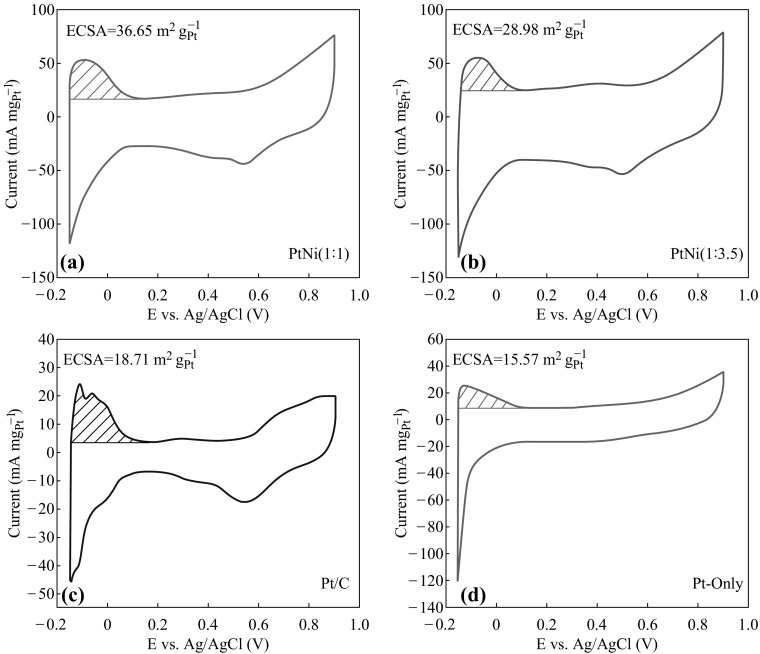
Cyclic voltammetry curves of **a** PtNi (1:1) NPs, **b** PtNi (1:3.5) NPs, **c** commercial Pt/C catalyst, and **d** Pt-only NPs, measured at room temperature in 0.5 M H_2_SO_4_ with a scan rate of 50 mV s^−1^

The electrochemical performance of the Pt/PtNi-NP-MWNT hybrids was characterized by the CV curves as shown in Fig. 5. The CV curves are featured with three parts: the hydrogen region, the double-layer region, and Pt oxidation–reduction region. Due to a more negative oxidation potential of Ni, no characteristic features of Ni oxidation–reduction are observed in the scan range [44]. The electrochemical surface area (ECSA) was calculated based on the desorption area of hydrogen (dash area in CV) [41, 45]. The ECSA values for the Pt/C, Pt-only, PtNi (1:1), and PtNi (1:3.5) catalysts are 18.71, 15.57, 36.65, and 28.98 m^2^ (g_Pt_)^−1^, respectively. Thus, the alloying of Pt and Ni is in favor of increasing ECSA, and Pt-to-Ni ratio turns out to be critical to the electrochemical performance.

**Fig. 6:**
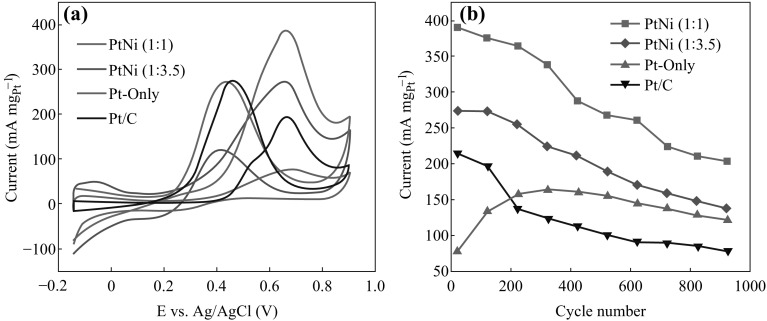
**a** Cyclic voltammetry curves of Pt/PtNi-NP-MWNT hybrids and commercial Pt/C catalyst, and **b** Pt-mass-normalized peak current versus cycle number, measured at room temperature in 1 M CH_3_OH + 0.5 M H_2_SO_4_ with a scan rate of 50 mV s^−1^

To evaluate the catalytic activity of the hybrids for methanol electro-oxidation, as depicted in Fig. 6a, the stable CV data were recorded in a N_2_-purged CH_3_OH + H_2_SO_4_ solution at a scan rate of 50 mV s^−1^. In the CV curves, two characteristic peaks are present in forward and backward scans, respectively. The ratio of the Pt-mass-normalized peak current in forward scan (*I*_f_) and that in backward scan (*I*_b_) is considered as a hint of the catalytic resistance to the CO poisoning, and a higher *I*_f_/*I*_b_ ratio may correspond to stronger CO poisoning tolerance [46–48]. The *I*_f_/*I*_b_ ratios of the Pt/PtNi-NP-MWNT hybrids are significantly higher than that of the commercial Pt/C catalyst, as summarized in Table 2. The Pt-only NPs have a maximum *I*_f_/*I*_b_ ratio; however, in this case the *I*_f_ value is very small presumably due to limited Pt active sites arising from surface oxidation. The surface oxidation of the Pt-only NPs will suppress the adsorption of methanol molecules and accordingly the CO poisoning as well.

**Table 2:** Electrochemical parameters of different catalysts calculated from cyclic voltammetry curve

Catalyst	Pt/C	Pt-only	PtNi (1:1)	PtNi (1:3.5)
ECSA [m^2^ (g_Pt_)^−1^]	18.71	15.57	36.65	28.98
Onset potential [V]	0.40	0.38	0.32	0.29
*I*_f_ [mA (mg_Pt_)^−1^]	198	80	390	274
*I*_b_ [mA (mg_Pt_)^−1^]	277	15	273	122
*I*_f_/*I*_b_	0.7	5.3	1.4	2.2

On the other hand, the onset potential for methanol electro-oxidation is another important parameter to evaluate the catalytic performance [49]. In forward scan, the onset potentials are 0.38, 0.32, and 0.29 V for the Pt-only, PtNi (1:1), and PtNi (1:3.5) NPs, respectively, all of which are lower than the one of 0.40 V for the commercial Pt/C catalyst. Significantly, the addition of Ni can effectively lower the onset potential, indicating the superiority of the PtNi alloy NPs for methanol electro-oxidation. As shown in Table 2, the PtNi (1:1) and PtNi (1:3.5) NPs show much larger *I*_f_ of 390 and 274 mA (mg_Pt_)^−1^, respectively, in comparison to that of Pt/C (198 mA (mg_Pt_)^−1^). The trend in *I*_f_ is well consistent with the ECSA results shown in Fig. 5.

According to the XPS and XANES results, the electronic structure of Pt was modified by the electron transfer from Ni, which will reduce the density of unoccupied states in the Pt *d*-band and hence weaken the Pt–CO bonding. In addition, the oxygen-containing species, which may be produced from Ni(OH)_2_, can react with adsorbed CO on the Pt surface [50]. This process will promote CO oxidation and then liberate the Pt surface from CO adsorption. Therefore, the PtNi alloy NPs show much higher catalytic activity than the Pt monometallic counterpart. However, excess Ni would cover the surface of Pt active sites and thus decrease the catalytic activity, such as in the case of the PtNi (1:3.5) NPs compared with the PtNi (1:1) NPs. As a consequence, an appropriate Pt-to-Ni ratio is needed to reach the highest catalytic performance. For instance, the PtNi (1:1) NPs are considered as having sufficient active sites and strong resistance against the CO poisoning at the same time.

Figure 6b shows a comparison in *I*_f_ of the studied catalysts in multiple CV cycles. After over 900 CV cycles, *I*_f_ of the PtNi (1:1) and PtNi (1:3.5) NPs maintain 52.1 and 50.5 % of their initial ones, respectively. In contrast, *I*_f_ of the commercial Pt/C catalyst maintains only 36.6 % of its initial one. The results demonstrate the long-term stability of the PtNi-NP-MWNT hybrids. As for the Pt-only NPs, *I*_f_ is increased first and then decreased with raising the cycle number. The origin of the *I*_f_ increase in the beginning is unclear so far, whereas it is worth noting that *I*_f_ for the Pt-only NPs are always smaller than those for the PtNi alloy NPs in all the cycles.

## Conclusion

The PtNi alloy NPs decorated on MWNTs were successfully prepared by the RTIL-assisted sputtering method with a PtNi alloy target. The PtNi alloy NPs exhibit small size and uniform distribution, and significantly Pt-to-Ni ratio can be controlled by selecting an appropriate alloy target. The PtNi-NP-MWNT hybrids are demonstrated to have high catalytic activity and long-term stability for methanol electro-oxidation, which are far superior to both the Pt-only monometallic counterpart and the commercial Pt/C catalyst. The XPS and XANES results indicate the electron transfer from Ni to Pt in the PtNi NPs, and it is beneficial to reducing the CO poisoning on the Pt surface. The present approach is promising for simple preparation of alloy-based nanocatalysts used in high-performance DMFCs.

## Electronic supplementary material

Below is the link to the electronic supplementary material.

Supplementary material 1 (PDF 237 kb)
