# Supplementary material for: Self-Decoration of PtNi Alloy Nanoparticles on Multiwalled Carbon Nanotubes for Highly Efficient Methanol Electro-Oxidation
Source: Nanomicro Lett. 2016 Jul 1;8(4):371–80. doi: 10.1007/s40820-016-0096-2 (PMC6223686; doi:10.1007/s40820-016-0096-2)
Supplement: Supplementary file 1 — Supplementary material 1 (PDF 237 kb) [file 40820_2016_96_MOESM1_ESM.pdf]

Supporting Information for

Self-Decoration of PtNi Alloy Nanoparticles on Multi-Walled Carbon Nanotubes for Highly Efficient Methanol Electro-Oxidation

Yu-Yan Zhou[1], Chang-Hai Liu[2,*], Jie Liu[1], Xin-Lei Cai[1], Ying Lu[1], Hui Zhang[1], Xu-Hui Sun[1], Sui-Dong Wang[1,*]

[1]Institute of Functional Nano & Soft Materials (FUNSOM), Jiangsu Key Laboratory for Carbon-Based Functional Materials & Devices, Soochow University, Suzhou, Jiangsu 215123, People's Republic of China

[2]School of Materials Science & Engineering, Changzhou University, Changzhou, Jiangsu 213164, People's Republic of China

* Corresponding author. E-mail: wangsd@suda.edu.cn, liuch@cczu.edu.cn

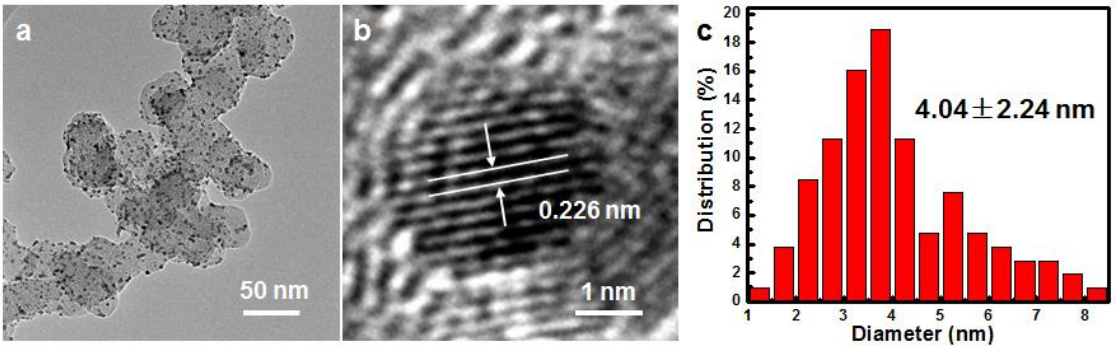

Fig. S1 **a** TEM image, **b** HRTEM image, and **c** NP size distribution of commercial Pt/C catalyst
